# Space Biomedicine: A Unique Opportunity to Rethink the Relationships between Physics and Biology

**DOI:** 10.3390/biomedicines10102633

**Published:** 2022-10-19

**Authors:** Mariano Bizzarri, Valeria Fedeli, Aurora Piombarolo, Antonio Angeloni

**Affiliations:** 1 Department of Experimental Medicine, University La Sapienza, 00161 Rome, Italy; 2 Systems Biology Group Lab, Dip. “P.Valdoni”, University La Sapienza, 00161 Rome, Italy

**Keywords:** space biomedicine, microgravity, non-equilibrium thermodynamics, systems biology, microenvironment

## Abstract

Space biomedicine has provided significant technological breakthroughs by developing new medical devices, diagnostic tools, and health-supporting systems. Many of these products are currently in use onboard the International Space Station and have been successfully translated into clinical practice on Earth. However, biomedical research performed in space has disclosed exciting, new perspectives regarding the relationships between physics and medicine, thus fostering the rethinking of the theoretical basis of biology. In particular, these studies have stressed the critical role that biophysical forces play in shaping the function and pattern formation of living structures. The experimental models investigated under microgravity conditions allow us to appreciate the complexity of living organisms through a very different perspective. Indeed, biological entities should be conceived as a unique magnification of physical laws driven by local energy and order states overlaid by selection history and constraints, in which the source of the inheritance, variation, and process of selection has expanded from the classical Darwinian definition. The very specific nature of the field in which living organisms behave and evolve in a space environment can be exploited to decipher the underlying, basic processes and mechanisms that are not apparent on Earth. In turn, these findings can provide novel opportunities for testing pharmacological countermeasures that can be instrumental for managing a wide array of health problems and diseases on Earth.

## 1. Introduction: Does Space Research Really Deserve Funding?

A few years ago, a report published by EMBO raised a number of doubts about the usefulness of research—especially biomedical research—performed onboard the International Space Station (ISS) [1]. According to this report, “the results from the ISS laboratories are neither scientifically relevant nor applicable to use on Earth […]. The feeling that research performed on the orbiting laboratory has been rather poor, and not in line with the expectations of the scientific community, has risen to an alarming level”. The report goes on, arguing, “the case for sending people into space is getting weaker with advances in automation and robotics”, and, therefore, “research in space thus needs to evolve to focus more on what science needs”. Undoubtedly, some fields of investigation such as microgravity crystallization have not attained the expected achievements, and some think that the impact of microgravity-based studies has been quite disappointing, given that new ground-based technologies allow for results to be obtained that exceed what is obtained from research using space-based materials [1]. Moreover, even the advocated “lack of a comprehensive strategy to maximize the benefits in light of the substantial costs” is not groundless. However, these judgments are largely ungenerous as they minimize a number of relevant achievements obtained in the medical field. Furthermore, the quoted viewpoint is severely biased by the fact that it misses considering novel perspectives disclosed by the development of the ARTEMIS program. The ARTEMIS program [2] focuses specifically on organizing a set of intertwined scientific and technological research programs with the aim of returning to the Moon and establishing a permanent human settlement on the lunar surface. To address such a challenge, proper medical and technical countermeasures are needed to mitigate health hazards secondary to prolonged exposure to both radiation and weightlessness.

## 2. Why Is Space Research Worth Pursuing?

Indeed, in replying to the issues raised by the EMBO’s report, we should move on from simplistic statements that posit that biomedical space research aims to “prepare astronauts for long-duration missions farther into the solar system and provide lasting benefits to life on Earth”. Undoubtedly, human health is severely threatened by the space environment and the development of specific medical tools, and pharmacological countermeasures are both mandatory to ensure a minimum of safety conditions. 

**Figure 1:**
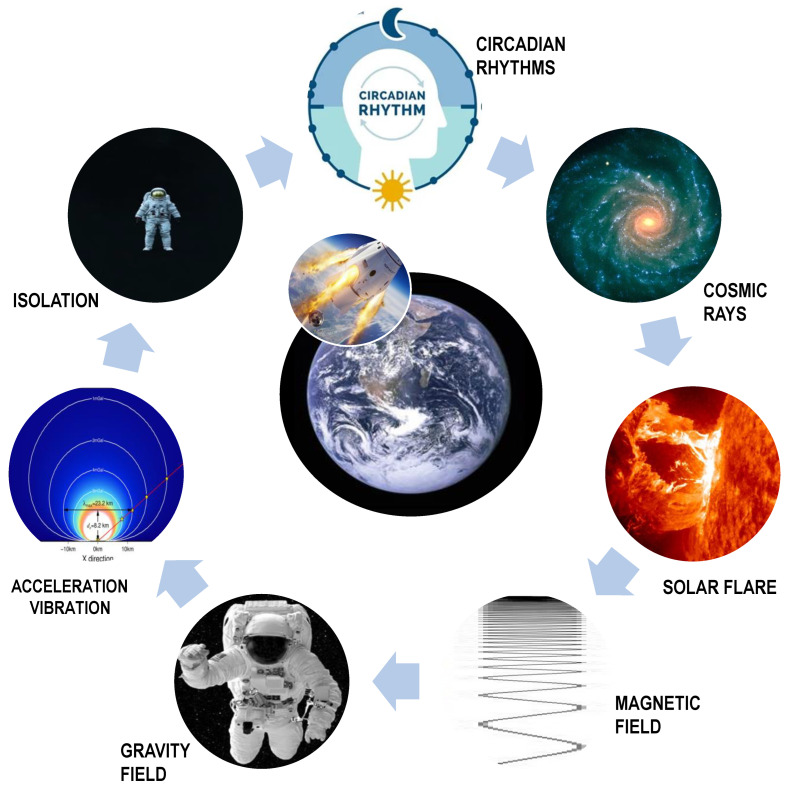
The space environment. Space is a hostile milieu due to dramatic changes in some critical, physical factors that can either directly or indirectly influence living functions. Consequently, people flying in space are exposed to significant changes in the gravitational and magnetic fields while being challenged by galactic cosmic rays and electromagnetic radiation as well as by uncomfortable conditions characterized by sudden acceleration modifications (comprising deceleration and vibration), physical and psychological confinement, and ultimately, severe disruption of basic chrono-biological rhythms.

The space environment is actually an “extreme” environment, in which humans are faced with three main, deadly challenges: (1) dramatic changes in fundamental physical forces (modified gravity and electromagnetic fields), affecting every level of the organism (from molecules to the entire human body); (2) exposure to cosmic rays and radiation hazards with a consequent increased risk of carcinogenesis and mutagenesis; and (3) endocrine and psychosocial upheavals following the physical isolation and disruption of fundamental chrono rhythms [3] (Figure 1). The overall health risk, albeit depending on the duration of the spaceflight and on the distance reached from the Earth, is, however, significant and cannot be underestimated [4]. 

Noticeably, our ignorance of the human physiological limitations severely limits our ability in planning future human exploration beyond the low Earth orbit (LEO), as those forecasted by the ARTEMIS program. The uncertainties cannot be restricted only to technological challenges, given that we are confronting the limits of medicine in addressing very unusual issues [5]. It has been argued that such limitations can be overridden by reducing human participation to a minimum, as robots can successfully replace humans, and science could be better served without a human presence [6]. The belief that robotic exploration is less expensive than human exploration and can display increased capabilities, thus exempting us from discovering appropriate countermeasures to ensure safe health conditions, further reinforces such assumption. However, several data provided by spaceflight missions, field analogue studies, and trends in robotic space exploration actually all point to exactly the opposite conclusion [7]. Furthermore, automated systems—sophisticated as they may be—are “restrained” in appreciating the “unexpected”. Ultimately, they look for what they have been designed to look for. In contrast, humans can actively cope with unexpected situations, which can finally result in priceless and serendipitous findings. Discovery is indeed an eminent creative process, a unique feature that robots still lack. Indeed, nobody can deny that human space flight is a unique kind of exploration that can expand the human (psychological and cognitive) experience, a goal that can never be achieved by a machine [8].

Definitely, the human capability in space is still irreplaceable to install and maintain complex scientific instruments and to conduct field exploration. These tasks take advantage of human flexibility, experience, and judgment. They demand skills that are unlikely to be automated within the near future, as a program of purely robotic exploration is inadequate in addressing the important scientific issues that make the outer space worthy of detailed study.

## 3. Progress in Basic and Applied Medical Knowledge

There are numerous cases of societal benefits provided by space exploration, from solar panels to implantable heart monitors, from cancer therapy to light-weight materials, and from water-purification systems to improved computing systems, and to a global search-and-rescue system, just to mention a few [9]. Moreover, it is hard to ignore that concerns for the safety and health oof astronauts have promoted a wealth of relevant discoveries in the realm of applied medical research. Undeniably, space biomedicine studies have fostered important achievements in different fields of research.

First, an impressive technological endeavor has been carried out to develop new diagnostic tools, medical devices, and infrastructures for developing remote sensing and medical support, given that medical capabilities are inherently limited during spaceflights [10]. Improvement in telemedicine and the realization of multi-sensor applications would never have been discovered if it had not been necessary for space exploration. Second, as the space environment poses dramatic threats upon specific apparatus including bone metabolism, cardiovascular system, muscle structure, and neurovestibular functioning, a systematic research on these systems has been pursued and some useful countermeasures have been recommended. Of note, some of these achievements can be conveniently introduced into clinical practice on Earth. Overall, these arguments pinpoint that space biomedicine has represented—and still represent—a major stimulus for the development of advanced biomedical technologies. Today, if we have new useful medical technologies—disposable plastic syringes, personal computers, new alloys for medical supports, improved analytical sensors and open magnetic resonance imaging (MRI), left ventricular assist device, light emitting diodes (LED, for surgical applications), pacemakers, bone densitometry, digital imaging breast biopsy system (developed from the Hubble’s telescope technology), laser applications, new tools for tissue culture, isothermal blanket, artificial prostheses, NeuroArm (a robotic arm that works on brain tumors in real-time), just to mention a few—it is thanks to space biomedical research [11]. Clearly, not only are these results of invaluable usefulness for ensuring an effective management of medical problems in space, but they also have greatly improved health care and diagnostic procedures on Earth. Remarkably, some specific technological applications have been proven to be instrumental in extending human control over the microbial environment in everyday life. For instance, the *Airin Space’s PlasmerTM bioprotection system*—a device that uses strong electrical fields and cold-plasma chambers to eradicate microorganisms in the spacecraft atmosphere—can safely protect the hospital, environmental, food, pharmaceutical products, and travelers against contamination/infection risks [12]. 

Similarly, the management of a number of different pathological and para-physiological conditions have benefited from the medical research carried out in space [13]. Osteoporosis, fracture management, intra-thoracic pressure regulation, lung deformation, de-regulation of sensorimotor, and neurovestibular function de-regulation are among the disturbances for which biomedical space research has provided relevant new insights and therapeutic breakthroughs [14]. Many of these scientific results have found their way into terrestrial medical applications. Disease treatments as well as life support for elderly and disabled individuals are currently being improved using medical devices and therapeutic procedures, which incorporate advanced technologies first developed for space flight applications. Finally, research performed in the very unusual outer space environment offers an unlimited horizon for investigation and discovery. Controlled studies aimed at investigating the effects of radiation, cosmic rays, or microgravity upon cells, tissues, and small living organisms—from bacteria to mice—have fostered the development of specific technological devices for more sophisticated 3D-cultures, which allow us to appreciate the critical role supplied by many different biophysical cues in biology. These conditions include shear stress [15], microfluidic-based approaches [16], cell colocation [17], organoid development [18], and environmental stiffness [19], just to mention a few. Overall, the investigation of cells/tissues growing in a modified physical milieu can serve as a novel paradigm for innovation, highlighting how architecture and physical interactions can efficiently shape the behavior of living structures. This kind of study has been proven particularly fruitful in the cancer field, and it has opened up new perspectives in both basic and clinical research [20].

## 4. The Great Challenge: Unveiling the Relationship between Physics and Biology

Space biomedicine has promoted a priceless wealth of results in addressing issues that are related to biology and physiology in extreme environments. Nonetheless, we believe that its unique, pivotal value should be sought elsewhere. Undeniably, biological investigations carried out in a space environment have forced us to cope with a true conceptual revolution in which the relationships in between physics and biology should be reframed in full. 

This is probably the most exciting and promising field of enquiry, as these relationships entail a different theoretical viewpoint, and can no longer be restricted to technical/technological approaches aiming at studying biological phenomena with increased precision. However, merging physics with biology is not simply a matter of the better “quantification” of biological entities, as recommended by Lord Kelvin, who stated, “When you can measure what you are speaking about and express it in numbers, you know something about it; but when you cannot express it in numbers, your knowledge is of a meagre and unsatisfactory kind” [21]. Is quantification really enough? However, one is tempted to quote Oppenheimer, who in, turn quoted Gödel: “It is purely an historical accident that [mathematics] developed along quantitative lines” [22].

Indeed, the development of non-equilibrium thermodynamics [22], together with the emergence of new conceptual perspectives such as those provided by self-organization and complexity [23] has yielded unique opportunities for a conceptual rethinking of the theoretical basis of biology and medicine. Namely, these questions matter when we look at living entities as structures displaying “organized complexity”, according to the seminal intuition of Warren Weaver [24]. Organized complexity is the specific feature of living systems where several components establish dynamic relationships, which change over time, showing non-linearity, bi-stability, and hysteresis. The genome cannot manage the “directionality” of processes that are “governed” at a higher level, in conformity with the system-related, general laws [25]. As a consequence, the interplay of physical principles and constraints ultimately rules the way molecular entities participate in producing complex structures across morphogenetic and differentiating processes [26]. These phenomena occur at the mesoscopic scale [27]—an intermediate position between atomic and macroscopic dimensions—where entities interact, thus allowing for coherent “organization” to emerge. The coherent form adopted by a chemical cascade of an organized complex structure (e.g., the cytoskeleton (CSK)) is probably the foremost relevant feature that we should consider when we try to understand how a physical/biological “signal” is transduced. Particularly, changes in the cell shape do not only affect the mechanotransduction of several environmental cues, but also significantly interfere with many intra-cellular pathways, as cell-signaling cascades can be turned on and off, both locally and globally, in response to alterations in cell size and shape [28]. More generally, every condition that can trigger nonlinear reactions or promote symmetry breaking can significantly modify the biological process under scrutiny, undermining naïve deterministic assumptions based on a linear analysis of the biochemical network [29]. Investigations performed during the last 20 years in this area have provided convincing evidence and contributed prominently in reconsidering the relevance of physical concepts such as the “field” in biology studies, as anticipated by the pioneering works of S.H. Burr [30].

Many scientists believe that the design of the whole system (and its evolution over time) can be deduced from the complete description of all of the entities of which the system is composed. This viewpoint posits that the architecture of a living organism can be explained by analyzing its constituent chemicals and their interacting capabilities, usually studied in isolation. The alternative hypothesis, however, maintains that there are relationships between entities that not completely derivable from the nature of the entities themselves. This is especially the case for emergent properties that are at the core of complex behavior, as morphogenesis and cell fate commitment [31]. It is now widely accepted that the reductionist perspective is unable in offering a tenable explanation of these processes. In the last analysis, the relational forces emerging from the field in which living objects are embedded control the directions in which activities move, and which therefore impart a specific pattern to the arrangement of single molecules in edifying the overall structure. Noticeably, the epistemic value of the field’s concept allows us to explain the emergence of several properties observed in developing organisms such as coherence, the establishment of long-range correlations, and critical transitions across which the system differentiates and evolves [32]. 

*Fields in biology*. A physical field exists if a discrete value of a parameter—usually a physical quantity—can be assigned to a given spatial region within the total area designated by the field. In molecular biology, one is accustomed to describing the fields as patterns of molecular species within cells, and pattern analysis is a major preoccupation of biologists investigating cancer and developmental related problems [33]. In particular, a scalar field represents a field of scalar quantities such as temperature, molecule concentration, or even gravitational acceleration. Indeed, a scalar field of gravitational potential does exist in living organisms on Earth, and this potential depends on the relative distance between the top and the bottom of the structure considered, as gravity acceleration (*g*) decreases with the increased distance (*h*, height) of the system from the center of the Earth, according to the equation:

(1) $$\Delta g\;=\;-2\;\cdot \;g\;\cdot \;\frac{h}{R}$$

where *R* = distance from the Earth center. As a result, we can observe appreciable effects only if the gravity potential is established for *h* > 1 cm. Therefore, the gravitational potential displays a remarkable gradient in the orthostatic posture in humans, especially involving the dynamics of fluids within the body. These effects are ascertained at the macroscopic level and significantly affect many critical processes including hydrostatic pressure, convection, shear stress, and Rayleigh convection (buoyancy), while diffusion is not influenced by the gravitational potential [34]. Accordingly, cells and tissues may be able to sense gravity changes in an indirect fashion through the modification of these processes that significantly modulate a number of pathways, especially those involving the interplay in between the cells and their microenvironment [35]. In plants, some specific mechanisms for gravity sensing have been recognized [36]. However, neither specific mechanisms nor specialized cells/organelles have so far been identified in mammal cells. Therefore, given that gravity force is orders of magnitude weaker than those governing the macromolecular interactions, at the very beginning of space exploration, the sensitivity of living structure to gravity has been ascribed as an “indirect” response to the modified biophysical properties of the environment at the macroscopic scale.

*An approach based on the non-equilibrium thermodynamics*. There is still no science that can explain in principle how single cells can detect gravitational accelerations in the range of 0–1 g. Physics predicts that reduced gravity can hardly cause any relevant changes in cells because gravity is an extremely low force when compared to other physical forces acting within the cells. To provide some illustrative examples, it has been calculated that the gravity force is 4,000,000× smaller than the force exerted by surface tension. The force required to move even a single electron within the electrical field of a nerve membrane approaches the weight of the entire cell, while the contractile force of a sarcomere equals the weight of more than 50 cells [37]. Quantitatively, we can estimate the gravitational potential energy of a cell in 21 kJ mol^−1^, while the chemical energy of only one hydrogen bond accounts for 17 kJ mol^−1^. All of this evidence indicates that gravity would have no impact whatsoever when the molecular level is taken into consideration.

However, since the seminal paper authored by Pollard [38] in the sixties, it was demonstrated that living cells can efficiently “sense” and respond to changes in the gravitational field. Afterward, a compelling body of experimental evidence has demonstrated that even a small reduction in the gravity force—as that experienced onboard the ISS [39]—can severely interfere with several sub-cellular structures and biochemical pathways [40].

In particular, thousands of genes and even unrelated pathways showed dramatic changes, suggesting that the modified gravitational field can impinge on the whole system [41]. It is quite unlikely that such a scenario could be ascribed to the unleashing of a thousand “signals”, interfering with a corresponding number of distinct targets. In contrast, the modifications observed on gene activity are probably a secondary output of a generalized disturbance in the field that, in turn, compromises the overall system functioning [42]. 

To find an attractive solution to this issue, we have to adopt a very different framework, based on non-equilibrium thermodynamics, and novel conceptualizations such as those offered by systems biology [43] and the theory of self-organized complexity [44]. Therefore, instead of focusing on single targets that can be stricken by a (gravity) “force”, we should look at the general dynamics that govern the system.

To put the question in thermodynamic terms, the gravitational energy *Eg* of a gas can be expressed as

(2) Eg=mgl

where *m* = molecule’s mass, in a volume of length *l*, subject to a gravitational acceleration *g*. The ratio of the energy interaction between the field and the thermal energy is of course

(3) $$\frac{mgl}{kT}$$

where *k* is the Boltzmann constant and *T* is the absolute temperature. A gravity field of this kind can hardly produce significant effects, given that *kT* ≫ *mgl* for values *l* < 1 cm. However, when the system approaches a tipping point, the increased cooperativity among a cluster of components amplifies their respective fluctuations, allowing the external energy of the field to overcome the thermal fluctuation (kT). It has been experimentally verified [45] that in such conditions, the ratio between the energy field and the thermal fluctuation increases significantly from *mgl*/*kT* to *mgl*/*kT*^1/3^, being

(4) $$\frac{mgl}{kTe^{1/3}}\;\gg \;\frac{mgl}{kT}$$

Equation (4) means that far from equilibrium, the (weak) field force (gravity) can actually overcome the thermal molecular fluctuations and superimpose its effects upon the overall dynamics. The fractional exponent in Equation (4) can vary from 2 to 3, depending on the context, and it arises from the nonlinearity of the non-equilibrium thermodynamics. 

A basic difference between systems in thermodynamic equilibrium and those far from the thermodynamic equilibrium such as dissipative living systems is in the way they react to external factors and forces overall represented within the field in which the system is embedded. Classical thermodynamics (i.e., equilibrium thermodynamics) is based on an idealized conceptualization for which systems are viewed either as isolated or closed systems, given that they are insensitive to their environment. However, truly isolated systems are rare. Thus, classical thermodynamics often deals with systems that usually exchange energy with their environment. In contrast, living organisms are “open systems” as they exchange both energy and matter with the surrounding milieu [46,47]. This statement has huge theoretical consequences: (1) the state of the system is resilient with respect to “weak” perturbations that are damped, allowing the system to recover its equilibrium; (2) weak external influences such as those exerted by gravitational or low electromagnetic fields have no effects as their interaction energy is negligible when compared to the thermal energy of the system’s molecular components. Within the state of equilibrium (where entropy is maximized) no cooperativity does exist between the single components. In non-equilibrium systems, when the system approaches a bifurcation point prior to a phase transition, some parameters show a bewildering rise in both the variance and reciprocal correlation, leading to a dramatic (and sudden) increase in their connectivity. In a second step, observables that were unaffected at the very beginning of the process will be “collectively” hauled into the transition to finally enter into a new attractor [48]. This behavior emphasizes that the transition toward a new attractor cannot be ascribed to a change in the “signaling” capability of individual molecules ruled by linear dynamics. Instead, the energy required for supporting the transition comes from the interaction with the field (the environment) of a group of highly correlated and intertwined factors. As clearly stated by Kondepudi and Prigogine, “This happens when there is cooperativity between the molecules in bringing about a macroscopic order; in a way the effect of the field on each molecule adds up to produce a large effect. In equilibrium systems this happens during a phase transition [while] in non-equilibrium systems the same type of phenomenon becomes much more interesting due to the richness of instabilities and the consequent transition to very intricate dissipative structures” [49]. In this way, dissipative processes that in principle could act by destroying order begin to interact near the transition point, leading to cooperative effects that ultimately increase the overall coherence. In living organisms, accessing a new attractor fosters the production of novel structures that “shape” the new phenotype the system actually acquires. We would stress that the sensitivity to gravity (or to other weak forces) can arise only during those phase transitions that are governed by non-equilibrium thermodynamics, and this is why non-equilibrium thermodynamics should be considered as a pivotal actor in the development of biological entities [50]. 

Therefore, when the system undergoes a non-equilibrium process, even weak forces such as gravitational or electromagnetic can significantly influence the dynamic outcome. Indeed, we can appreciate the relevance of that mechanism when the system is placed in microgravity (i.e., when these very weak forces are “removed” from the field). Indeed, if we look at Equation (2), when the *g* values approach zero, then *mgl*~0, and the system’s component are exposed to only the thermal fluctuation, which would lead to a bewildering number of different configurations, each one being equiprobable. Incidentally, we should remember that Equation (4) is obviously valid even if *g* ≥1, thus suggesting that in hypergravity, thermal fluctuation (*kT*) is further damped. 

The equiprobability of different solutions means that a system in microgravity shows *symmetry*. The symmetry is reflected in the *multiplicity* of solutions: there are—at least—two possible dissipative structures to which the system can transition. A similar situation occurs, for instance, in the Rayleigh–Bénard convection. Above a critical temperature gradient, convection rolls emerge. Each individual roll can assume a clockwise or counter clockwise rotation: both of these states have the same probability, and the choice between them will depend on the influence exerted by external factors, which biases the system toward one of the two possible polarities [51].

### 4.1. Non-Equilibrium Thermodynamics and Pattern Formation

Systems far from thermodynamic equilibrium such as living ones can spontaneously self-organize into structures that exhibit chemical oscillations, propagating waves, and stationary chemical patterns. These non-equilibrium structures explain how emergence and novelty arises, and how these features can be understood from a thermodynamic point of view as critical characteristics of dissipative structures. A system near equilibrium spends a part of its time in a stationary phase, governed by a linear regimen of forces. This linearity is lost when internal/external processes perturb the system—as the relationship between thermodynamic flows and forces is no longer linear—and the system becomes unstable. Some variables undergo a progressive increase in their fluctuations that cannot be damped by irreversible processes (as happen in the stable state). In this condition, beyond a critical value of one or more parameters, the system approaches the point where it can make a transition toward novel stable-states (attractors). 

The sensitivity of dissipative systems to changes in the external field confers unique properties as these systems are subject to a regimen of extended critical transitions [49] (i.e., a succession of symmetry breaking events through which their intrinsic functions/structures become correlated with the surrounding field. Considering the number of transitions that a non-equilibrium system usually undergo, such “sensitivity” plays a very relevant role in governing the overall dynamics of the system, unlike what happens in equilibrium systems in which phase transitions are isolated occurrences. Moreover, this sensitivity plays a critical role during pattern formation and is definitely at the origin of novelty in biological evolution. Indeed, it can be argued that local and global symmetry changes along extended critical transitions characterize the behavior of living organisms and provide support for the intrinsic variability that represents the true, main invariant of the living state of matter [52]. These statements have a huge consequence for the origin and definition of life, and have been discussed in detail elsewhere [53]. In turn, pattern formation is at the core of very relevant processes in biology such as differentiation, morphogenesis, and even cancer transformation. In all of these conditions, when the system approaches a bifurcation point, which in principle would allow accessing different phenotypes/attractors, the critical role of the forces that are expressed by the field becomes prominent, and can drive the overall process into a deterministic outcome [54].

Pattern formation is not exclusive to living systems, as even ‘simple’ chemical systems such as those represented by the chemical oscillations and spiral waves produced in the Belousov–Zhabotinsky (BZ) reaction may exhibit it. Regarding the BZ reaction, notwithstanding the reaction consists of a “simple”, oxidant–reduction process in which an organic reductant (i.e., bromate) is oxidized by a redox couple, the process yields a very rich phenomenology of wave formation. Such structures maintain themselves through the dissipation of free energy generated during irreversible processes. Noticeably, the BZ reaction can exhibit deterministic chaos or other unpredictable behaviors and downward symmetry breaking such as those induced by gravity changes. The modulation of waves generated during the BZ reaction is ascribed to two distinct processes (i.e., convection and diffusion). As diffusion is not a process that can be influenced by gravity, it has been assumed that gravity could interfere with convection only. Indeed, preliminary studies showed that the wave propagation velocity under microgravity decreased to ~80% with respect to on ground experiments due to the disappearance of convection. When the BZ reaction was investigated within a gel matrix, no significant differences were found, since convection within a viscous milieu was almost completely absent even under normal gravity [55]. However, further detailed investigations have revealed that, in gels, the wave propagation velocity was also greatly reduced by microgravity, albeit less than in fluid systems [56]. These intriguing findings, obtained in a weightlessness regimen in which buoyancy, convection, and diffusion no longer play a role due to stirring, demonstrated that gravity should interfere with at least one of the rate constants of the chemical reactions underlying the system. 

Other chemical systems, when they are far from equilibrium, become very sensitive to even mild physical cues. For instance, when sodium chlorate (NaCIO_3_) molecules—deprived of optical activity—are left to crystallize from an aqueous solution, an equal number of levo (l) and dextro (d) NaCIO_3_ crystals can be obtained. However, if the solution is stirred, we obtain a sample in which almost all of the NaCIO_3_ crystals showed that they had the same chirality (either levo or dextro) [57]. This amazing result clearly indicates that upon the influence of a mild physical stimulation, a dynamic system can undergo symmetry breaking, as anticipated by non-equilibrium thermodynamics. While in resting conditions, the chemical reactivity of the above-mentioned system is solely ruled by thermal fluctuation of the molecules, the physical energy provided by stirring the solution can be enough in inferring a specific directionality to the evolving system. Therefore, only a single (optical) configuration arises. This example clearly shows that novel forms can “spontaneously” appear without the help of any “information” transduced by specific “molecular signals”. The “morphogenesis” of this process is ultimately the result of the non-equilibrium dynamic regimen that governs the interplay between the system and its microenvironment. We would like to emphasize this conclusion as it sheds light on the intricate matter of morphogenesis and differentiation in both evolution and embryology, explaining how the physical field can differentially “use” the same genetic information in producing very different structures. Overall, the aforementioned examples highlight how this kind of process, far-from non-equilibrium, can serve among others as a model for self-organization and pattern formation in biological systems including humans.

Indeed, there is widespread consensus on the fact that—after Turing [58]—spatial patterns produced by dissipative systems can help explain morphogenesis, as these structures are recognizable in living components, exhibiting oscillation and quasi-periodicity [59]. Moreover, the possibility that gravity changes could destabilize self-organization and pattern formation in living organisms may actually offer a useful experimental model for rethinking some basic concepts in theoretical biology. 

A homogeneous, quasi-stable system such as that constituted by living cells and tissues shows significant resilience with respect to mild perturbation emerging from the microenvironment. However, in some circumstances, such a system can undergo a dramatic transition toward a new stable state (the “attractor”) by relaxing into a new pattern of relationships in between the different components/structures of the system. This process entails a bewildering number of entities that—suddenly—display increased cooperativity and correlation between them, to ultimately collectively transit from an attractor to a new one. The usual explanation proposed by molecular biology resorts to the genome, claiming that a selected number of molecules provide the right “instructive” signals that will drive the process across sequential, ordered steps [60]. However, a compelling body of evidence has demonstrated that this reductionist stance is unable to explain the complexity of the phenomenon. Indeed, gene activity displays prominent stochastic features [61], and the subsequent random pattern of gene expression is likely to produce probabilistic outcomes, resulting in the coexistence of “alternative” phenotypes, even within the same system [62]. In this situation, because of the sensitivity to the initial condition, a small perturbation may grow exponentially with time, eventually pushing the system toward a chaotic state. However, this eventuality is unlikely to occur under ordinary conditions, given that the *field* provides the physical constraints that can prevent the emergence of chaotic behavior and finalize the biological process toward a specific, stable attractor.

These assumptions have been experimentally demonstrated by studies performed in microgravity with living cells. Animal and human cells [63,64] growing in weightlessness undergo relevant morphologic changes, as they partitioned—almost equally (~50%)—into two distinct phenotypes: adherent and floating cells. Intriguingly, the two phenotypes promoted by microgravity exposure can recover their native phenotypic morphology in a few hours when replaced into normal gravity. In fact, in 1 *g*, the two cell phenotypes collapse into one, indistinguishable from the original, ground-based phenotype. Surprisingly, when the two cell clusters previously obtained during a first-course culture in weightlessness were isolated, and then again separately reseeded in microgravity, the two distinct phenotypes—adherent and floating cells—emerged once more from each cell cluster. As a result, cells cultured in microgravity endlessly “travel” from one phenotype to another. Conversely, only in the presence of gravity, the system “accesses” into a stable attractor: the “weak” gravitational field therefore becomes irreplaceable during cell fate commitment. Noticeably, these phenotypic changes go together with a coordinated motion in the gene-expression phase-space without entailing major changes in gene activity. These results suggest that the emergence of different phenotypes can be sustained by the same genotype, which preserves its overall coherence across the critical transition. 

**Figure 2:**
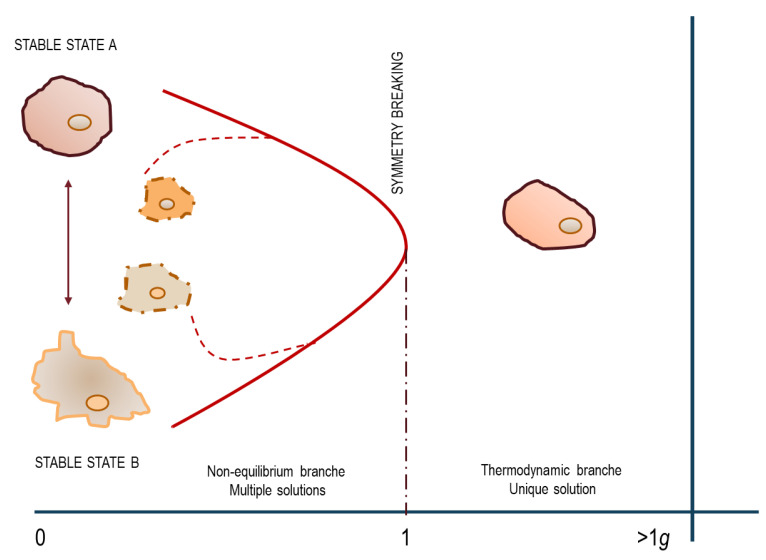
The diagram depicts a system travelling across symmetry breaking, thus alternatively accessing two different regimens, featured by equilibrium and non-equilibrium thermodynamics, respectively. Within the gravity field, the system displays a linear behavior and thermodynamics allows only one stable solution: the fate commitment of the cell(s) is unambiguously (and deterministically) recognizable. Downstream of the bifurcation point, in microgravity, the system recovers its symmetry. The previous thermodynamic state becomes unstable and new configurations arise, some of which are transitory, being unstable or meta-stable. This experiment (adapted from [61]) demonstrated that the presence of even a weak force such as gravity can exert a significant effect upon the evolution of a non-equilibrium process by constraining that evolution into a unique stable, configuration.

This experimental result vindicates the theoretical explanation anticipated by Kondepudi and Prigogine [49]: a system distributed in two distinct attractors collapses into a unique configuration when exposed to gravity that ultimately “selects” from two potentially, accessible states (Figure 2). These results substantiate the occurrence of a symmetry-breaking transition. A symmetry-breaking transition is such that in the absence of an external driving force such as gravity, each of many possible final states can be reached with equal probability. This simple experiment demonstrated that a field ‘deprived’ of the gravity “force” cannot provide stable conditions, as weightlessness favors a never-ending transition among different phenotypes, thus enabling the establishment of a ‘permanent transition state’, where the “extended criticality” allows the system to travel across unlimited phase transitions [65]. Conclusively, in the absence of proper physical constraints including gravity, temperature [66], or electromagnetic fields [67], cells are unable to find a unique, specific differentiated fate [68]. 

Overall, these findings substantiate the logic behind the “emergentism” proposed by Michael Polany. In Polany’s perspective [69], molecular (protein and genetic) properties are constrained by higher-level ordering principles [70], thus leading to a huge set of events, as recently demonstrated in in silico models [71]. Within that framework, boundary conditions (i.e., constraints) supply degrees of freedom that, instead of being random, are determined by higher-level realities, whose properties are dependent on but distinct from the lower level from which they emerge.

However, living organisms including molecular structures such as proteins can actually display only a very restricted number of configurations (“forms”), precisely those allowed by the laws of a higher order, not implicit in the lower one [72]. Thereby, the laws of higher order ultimately determine the system’s behavior, while the laws governing the microscopic realm only provide the necessary condition for its functioning [73]. These findings show that different ordered structures (i.e., stable phenotypes) could exist in equilibrium, depending on the environmental conditions, their similar genotype notwithstanding. Examples provided by studies in the space environment have demonstrated that organisms actually “use” the field’s forces (e.g., gravity) to harness the intrinsic stochasticity of lower levels, thus ultimately producing order, making those choices that offer the fittest solution to environmental challenges [74]. This finding undermines the concept of the genetic program such as that borrowed from information theory and electronic computers [75]. Simply said, undoubtedly, morphogenesis and cell fate commitment are both under strict genetic control. However, genes do not produce phenotypes and forms by themselves, while physical mechanisms do [76]. Therefore, biochemical and epigenetic processes associated with gene expression patterns must set up the *permissive* conditions for the physical mechanisms, which in turn *constrain* the outcome of biological structure formation [77,78].

### 4.2. Microgravity and Mechanomics

It is noticeable that non-equilibrium thermodynamics severely affects the intracellular structures that are primarily involved in mechanomics. The term “mechanomics”, first introduced by Sem et al. [79], describes the mechanical phenomena involved in cell physiology. Specifically, mechanomics refers to the mechano-transduction inside the cell(s) of many environmental-produced forces that, in turn, translate their impact into biochemical/genetic machinery changes.

A wide array of physical forces (shear stress, surface tension, tensile, and compressive forces, etc.) emerging from the microenvironment, due to the agency of several stroma components included within the overall physical field, dynamically interact with specialized cellular structures, principally the cytoskeleton, which are instrumental in transmitting the force effects into the cells up to the nucleus. 

The cytoskeleton constitutes a pivotal structure that confers shape and stability to the cell, while serving as sensor of mechano-biological inputs from the microenvironment. Noticeably, through the remodeling of CSK, mechanical forces can be transduced even to the organelles and nucleus, thus influencing the structure of the nucleoskeleton, and furthering the gene expression pattern [80]. Thereby, CSK is currently viewed as a complex “sensor” in which external and internal stimuli both converge. CSK adaptive changes may either amplify or dampen several kind of physical stresses, hence triggering a collective, sand-pile like response of many intracellular components [81]. The CSK plays an instrumental role in supporting cell division, migration, invasion, and differentiation in response to biophysical and thermodynamic stresses, rather than to a single molecular input [82]. It is noteworthy that CSK is itself a non-equilibrium chemical system with the capability to harness chemical energy to perform mechanical work. Indeed, the functioning of CSK occurs within a regimen on endless remodeling, involving a continuous disassembly and assembly of the monomer constituents. This process is highly unstable, and the resulting stationary macroscopic pattern is significantly sensitive to biophysical perturbations including gravity [83]. Noticeably, cytoskeletal proteins are influenced by microgravity at a very early stage, given that the CSK network resulted in being perturbed a few seconds after the exposure to weightlessness [84] before any recorded change in the gene expression or biochemical cascades [85]. 

The gravitational field acts by “canalizing” monomer movements, allowing the emergence of striped patterns of microtubules oriented consecutively in an ordered manner. However, in weightlessness, monomer alignment is perturbed by thermal fluctuations and consequently no pattern formation arises, and microtubules self-organize into an isotropic configuration without preferential orientation. These findings clearly demonstrate that the gravitational field imposes a vector of directionality to the self-organizing process by inducing symmetry breaking upon the non-equilibrium thermodynamics, which rule the overall behavior of the system [86]. In the absence of gravity, the field showed no asymmetry and the CSK could freely access different “attractor” states. In contrast, when gravity is restored, the perturbation of the bifurcation induced by the field allows the system to capture the external asymmetry and build patterns of preferred polarity or preferred chirality [87]. In this way, the weak force represented by gravity can yet “constrain” the phenotypic configuration by “canalizing” the cytoskeleton and negatively selecting the shapes that are unfit to grow within the field. Conversely, when this constraint is reduced or disappears, a descent with modification yields a larger variety of enabled structures [88]. The resulting phenotypes emerging from the field are therefore due to the intrinsic plasticity of organismal development, supported by their inherent stochasticity in gene expression. Cytoskeleton proteins indeed behave as a true dissipative system, and the CSK architecture can “capture” and further amplify even minor changes occurring in the field of forces, precisely because the non-equilibrium dynamics governs CSK activity and conformation. Ultimately, this mechanism allows cells to display emergent properties, long-range correlations, and dramatic first-order class transitions, which play a pivotal function in a number of relevant biological processes including proliferation, differentiation, embryo-, and morphogenesis [89]. Consequently, non-equilibrium thermodynamics can significantly impair the overall mechanomics of the system, thus contributing further to the changes observed in all of the conditions in which the living system undergoes a critical transition.

## 5. Perspectives and Conclusions

Studies performed in microgravity are going to disclose exciting new areas of investigation, and can promote the development of novel products for the benefit of humankind. Adaptive capabilities of plants and crops as well as the emergence of new “varieties” showing higher nutritional values, yields, and critical advantages (e.g., resistance against high and low temperatures, salt stress, microbial and pest attacks) can be conveniently exploited to improve food production [90]. Growing plants or unicellular organisms such as fungi and algae in a microgravity environment could reactivate either dormant genes or promote a different gene expression pattern, supporting the synthesis of unexpected products such as drugs [91]. Thereby, the space environment offers a unique opportunity to investigate how physical stressors can drive evolutionary processes toward a sought-after end by accelerating the search for adaptive phenotypes [92].

Space biomedicine has provided significant technological breakthroughs by developing new medical devices, diagnostic tools, and health-supporting systems. Many of these products are currently in use not only onboard the ISS, but have been translated into clinical practice on Earth. 

However, we believe that the most relevant and exciting contribution consists of the fact that studies investigating the properties and behavior of (living) matter in extreme physical environments have prompted a rethinking of the theoretical basis of biology. Overall, the studies herein reviewed have stressed the critical role that biophysical forces play in shaping the function and pattern formation of living structures. Investigating biological processes under microgravity conditions allows us to appreciate the complexity of living organisms through a very different perspective. According to these studies, biological entities should be conceived as a unique magnification of physical laws driven by local energy and order states overlaid by selection history and constraints, in which the source of the inheritance, variation, and process of selection has expanded from the classical Darwinian definition. Moreover, the very specific nature of the field in which living organisms behave and evolve in a space environment may provide a useful experimental model for deciphering underlying, basic processes, and mechanisms that are not apparent on Earth. In turn, these findings can provide novel opportunities for testing pharmacological countermeasures that can be instrumental for managing a wide array of health problems and disease on Earth.

Particularly, microgravity research emphasizes the *epistemic relevance* of the field concept in biology. Forces and constraints acting within a field superimpose their agency over the stochasticity displayed by molecular and genetic factors, which, by itself, lack the possibility to direct biological processes toward a deterministic outcome. Indeed, the “removal” of the gravitational constraint allows for the appearance of unexpected, different configurations, which emerge from a repertoire provided by the stochastic gene activity. This is likely to be a very general mechanism through which novelty is “produced” in biology: the field selects between alternative phenotypes spontaneously supported by the agency of alternative gene-regulatory networks [93]. Forces within the field—electro-magnetic, mechanical, and even gravitational forces—in association with physical and geometric constraints, select and then stabilize only a specific pattern among many. In the absence of forces and constraints provided by the field, living structures ruled by non-equilibrium thermodynamics could yield bi-stable and bi-modal decisions from noise and “intrinsic” genetic stochasticity [94].

This behavior is in agreement with the principle of “reaction norm”, which posits that the genotype can enact the emergence of several, equi-probable phenotypes that are chosen and stabilized by the microenvironment in a second step. Within that framework, the genome displays a “permissive” role, while the environment has an “instructive” function [95]. Noticeably, non-equilibrium thermodynamics help explain how even (apparently) weak forces can have dramatic effects, contributing as true causative factors in determining critical biological processes such as morphogenesis, differentiation, and cell fate commitment [96]. Experiments in microgravity allow for a unique and elegant opportunity to study in “isolation” the effects of at least one physical force (i.e., the gravity). Lack of gravity is an exceptional case, and it is obviously improbable that it could happen on Earth. However, that model can actually apply for several other forces—mechanical and electro-magnetic—acting through and within the microenvironment in which cells are embedded. Intriguingly, changes in the structure and composition of the microenvironment can modify the field of those forces—especially the mechanic ones—and in this way, the milieu can influence the fate of cells. As outlined by S. Huang, physical forces and constraints therefore play “a central role in how Gene Regulatory Networks produce, almost for free, the stable gene expression patterns needed to govern coherent cellular behaviors” [97]. We assumed that this evidence can no longer be ignored, still talking about the “deterministic” instructive role played by the genome in cellular processes. 

These findings have relevant theoretical consequences. (1) Modifications in the balance of forces acting within the microenvironment play a critical role in modulating critical transitions during the biological processes. Focusing upon a few molecular pathways could hence not be enough to explain the complexity ruling a number of biological properties such as differentiation, morphogenesis, and cancerous transformation, just to mention a few. (2) Phenotypic commitment should be viewed as a potentially *reversible* process, at least in some cases. Studies in which somatic differentiation can be successfully reverted/re-oriented through biophysical cues can offer heuristic examples [98,99]. Furthermore, as manipulation of the microenvironment has been demonstrated to be instrumental in reprogramming even cancerous cells [100], the possibility of field modification to attain a positive treatment result should be explored in depth. Consequently, the field (i.e., the microenvironment) is becoming a new and attractive target for pharmacological investigation across a wide range of different pathological conditions [101,102].

Furthermore, by replacing living systems within their proper environmental context (i.e., the physical field), we have the opportunity for a conceptual revolution in which theoretical approaches such as those offered by self-organized complexity and non-equilibrium thermodynamics theories can successfully help explain processes that can hardly be understood according to a reductionist stance. For instance, it is deplorable that the contribution of non-equilibrium thermodynamics to theoretical biology remains largely underestimated by the scientific community. Non-equilibrium behavior is ubiquitous and comprises both living and nonliving entities. The breadth of the phenomena investigated makes the study of non-equilibrium systems an inherently interdisciplinary field that forges connections between the physics community and researchers in biology and chemistry as far-from equilibrium physics underlies a wide range of phenomena outside the traditional boundaries of condensed matter physics. However, the perspective provided by non-equilibrium thermodynamics cannot be considered either as a simple extension of equilibrium (or near-equilibrium) physics, or as an additional “tool” to the “omics” disciplines that are currently evoked to handle the overwhelming body of data provided by investigations focused at the microscopic level. Indeed, the essential conclusion from the studies herein discussed is this: to describe the collective behavior of complex systems, we need entirely new concepts compared to the microscopic description. 

Definitely, non-equilibrium thermodynamics does not require achieving a “complete” knowledge of the bewildering multiplicity of the interactions that take place at the microscopic level. The self-organization of patterns and structure formation arises from many nonlinearly interacting subsystems, depending on the external control parameters—forces and constraints—provided by the field [103]. Biological research performed during spaceflight missions can provide a unique opportunity to test and understand this novel framework. Moreover, breakthroughs in this area of research promise to reframe the theoretical foundation of biology and achieve a far-reaching impact on many disciplines including clinical treatment.

Several key questions are still unanswered. Is the microgravity-related effect on living organisms truly irreversible? Can an adaptation of some sort be envisaged for long-duration space flights? Is there a threshold value for microgravity effects? Undoubtedly, a general theory about the relationships between gravity and life is needed. Understanding the relationships between gravity and life can also help understand how physical constraints are mandatory to allow life to develop. Those factors—temperature, pressure, salinity, radiation exposure and gravity—are among the commonalities of known Earth life, and should be quantified for different planetary contexts. Specifically, the question is whether life processes can tolerate a different regimen of microgravity. Specifically, studies are required to investigate whether low microgravity levels—albeit not negligible such as those recorded on the lunar or Mars surface—can be “compounded” to finally “support” living organisms, as observed in extremophiles to develop multiple adaptation strategies [104]. Indeed, many physical factors including temperature, salinity, and pressure can fluctuate in a wide range of values; nonetheless, several kinds of living organisms can survive and adapt to these unusual environments [105]. Can the same be true for the gravitational field? Namely, can living organisms undergo normal morphogenesis and growth in microgravity, thus allowing for the wide spread of life on planets characterized by g < 1? To address such an issue, we are planning an experiment that will be performed onboard the International Space Station to evaluate the functionality of ovary cells and their adaptive capacity in sustaining the reproductive process at their very beginnings. Further studies aimed at investigating the regularity of embryo development will provide significant answers.

## Data Availability

Not applicable.
